# Measuring novelty in science with word embedding

**DOI:** 10.1371/journal.pone.0254034

**Published:** 2021-07-02

**Authors:** Sotaro Shibayama, Deyun Yin, Kuniko Matsumoto

**Affiliations:** 1 School of Economics and Management, Lund University, Lund, Sweden; 2 Institute for Future Initiative, The University of Tokyo, Tokyo, Japan; 3 National Institute of Science and Technology Policy, Tokyo, Japan; 4 School of Economics and Management, Harbin Institute of Technology, Shenzhen, China; 5 World Intellectual Property Organization, Geneva, Switzerland; Universita degli Studi di Foggia, ITALY

## Abstract

Novelty is a core value in science, and a reliable measurement of novelty is crucial. This study proposes a new approach of measuring the novelty of scientific articles based on both citation data and text data. The proposed approach considers an article to be novel if it cites a combination of semantically distant references. To this end, we first assign a *word embedding*–a vector representation of each vocabulary–to each cited reference on the basis of text information included in the reference. With these vectors, a distance between every pair of references is computed. Finally, the novelty of a focal document is evaluated by summarizing the distances between all references. The approach draws on limited text information (the titles of references) and publicly shared library for word embeddings, which minimizes the requirement of data access and computational cost. We share the code, with which one can compute the novelty score of a document of interest only by having the focal document’s reference list. We validate the proposed measure through three exercises. First, we confirm that word embeddings can be used to quantify semantic distances between documents by comparing with an established bibliometric distance measure. Second, we confirm the criterion-related validity of the proposed novelty measure with self-reported novelty scores collected from a questionnaire survey. Finally, as novelty is known to be correlated with future citation impact, we confirm that the proposed measure can predict future citation.

## Introduction

Novelty constitutes a core value in science, as new discoveries shape the basis of scientific advancement [1, 2] and has broader impact on technological innovation [3]. Accordingly, novelty serves as a key criterion for the evaluation of scientific output as well as decision makings such as funding allocation, employment, and scientific awards [1, 4–6]. It is therefore critical that scientific novelty can be reliably measured. In practice, novelty is usually assessed through peer review on a small scale [7], while evaluating novelty on a larger scale remains to be a challenge. Though recent bibliometric techniques have enabled us to measure various qualities of scientific discoveries, including novelty [8–11], the validity and practical utility of the extant measures are debatable [12, 13].

Previous bibliometric measures for the novelty of scientific documents draw on roughly two data sources, either citation data or text data. Text data are of obvious use, in that once a scientific discovery is documented, its novelty should be revealed in text information. Nonetheless, due to the ambiguity and complexity of natural languages, previous measures use text data rather superficially without sufficiently exploiting the semantic information [e.g., 14]. It is relatively recently that such semantic information got extracted from text data and translated into bibliometric indices [e.g., 15]. To circumvent the technical challenges in extracting semantic information from text data, citation data have been extensively utilized in previous novelty measures. As a citation represents information flow from a cited document to a citing document, it can be used to infer certain qualities, including novelty, of a document without scrutinizing the content [10, 16]. However, the validity of this approach has been occasionally questioned [12]. In fact, insufficient validation has been a limitation common to most novelty measures [17]. Furthermore, a practical limitation common to previous measures is that they require access to a large-scale bibliometric database (often the whole universe of scientific documents), which are usually proprietary and expensive, and high computational power, which potential users of the measures do not always have.

**Fig 1:**
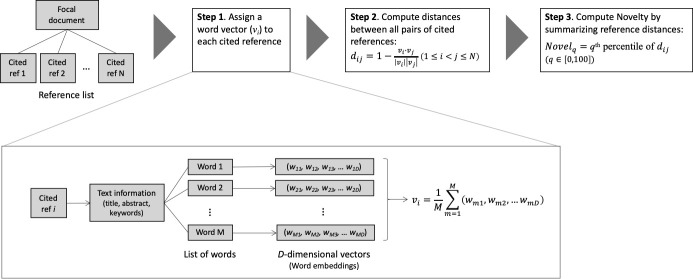
Algorithm of novelty computation.

To address previous limitations, we propose a new approach to compute the novelty of scientific documents by combining both citation and text data (see Fig 1). Our approach features *recombinant* novelty [18–21], considering a document to be novel if it cites a combination of semantically distant documents. This is in line with the previous measures based on citation data [e.g., 8]. Unlike previous measures, however, we use text data to quantify the distances between cited documents. Specifically, based on the text information included in cited documents, we map each document to a *word embedding*–a high-dimensional vector assigned to each vocabulary [22]–with which to compute distances between cited documents. To the best of our knowledge, this is the first to use the word-embedding technique to measure the novelty of scientific documents.

For text information, we test three sources–the abstract, keywords, and the title of cited documents–finding all satisfactory performance. Of the three sources, titles of cited documents are often included in the focal document itself, and the burden of data access is minimized. As a library of word embeddings, we draw on *scispaCy* [23], which is publicly available and thus significantly reduces the computational cost. We publicly share the code [24], with which one can compute the novelty score of a document only with the focal document’s reference list.

We validate the proposed measure in three exercises. First, we confirm that word embeddings from the selected library can be used to quantify semantic distances between documents by comparing with an established bibliometric distance measure. Second, we test the criterion-related validity of the proposed novelty measure based on self-reported novelty scores collected from a questionnaire survey. Third, as novelty is known to be a predictor of future citation impact [8, 11], we test whether the proposed measure is correlated with future citation.

This paper is structured as follows. In the next section, we categorize previous novelty measures and discuss their characteristics and limitations. The following section describes our proposed measure and outlines its operationalization. Then, we present the methods and data for the validation exercises. Finally, we present the results and conclude.

## Literature review

Previous bibliometric measures for novelty can be categorized based on their conceptualization and operationalization (Table 1). Conceptually, some measures aim to represent the uniqueness of a certain knowledge element (Groups 1 and 4)–for example, a discovery of a new molecule and development of a new material. In contrast, other measures aim to capture a recombination of knowledge elements (Groups 2 and 3), in which a new or rare combination of knowledge is considered to be a sign of novelty. The notion of recombination as a source of novelty has been widely discussed in the literature. The creativity literature argues that associating remote elements is a path to creative solution in general as well as in science [18, 19], and the management literature suggests that combining components is a major route to technological innovation [20, 21].

**Table 1:** Previous novelty measures.

Group	Description	Concept		Data	
		Recombination	Uniqueness	Citation	Text
1	A new word [14, 25*, 26*]		X		X
2	A new combination of words [9, 25*, 27*]	X			X
3	A new combination of cited references [8, 10, 11, 16*, 17]	X		X	
4	A distant text [15*]		X		X

Note.

*patent measures

For operationalization, a group of measures exploits citation information to assess novelty indirectly (Group 3), and the other draws on text analysis to assess the content of documents (Groups 1, 2, and 4). Among the latter, the majority uses text information only superficially without using the semantic information of the text (Groups 1 and 2), but recent measures attempt to extract semantic information (Group 4). Studies on novelty measures have been relatively advanced in technology management, in which a patent is used as a unit of document [e.g., 16, 25]. We also refer to these measures because the key idea behind the measures is applicable to scientific documents. In what follows, we discuss four groups of previous measures.

### (1) A new word

The first group of novelty measures is based on the first appearance of a word(s) that appears in a document [14, 25]. If a document includes or is associated with a certain word or a sequence of words that is new to the world, it can be inferred that the document delivers novel information. For example, if a document contains a previously unknown chemical compound, it suggests that the document is novel. In this category, Azoulay *et al*. [14] drew on Medical Subject Heading (MeSH), a controlled keyword dictionary, and operationalized the novelty of a journal article based on the average age of keywords (the number of years since its first appearance). Balsmeier *et al*. [26] and Arts *et al*. [25] also identified novel inventions based on the first occurrence of a word as well as a sequence of words (bigram and trigram) in patent documents.

### (2) Recombination of words

The second group is technically similar to the first group but conceptually different as it is to measure "recombinant" novelty [19, 20]. When a document includes a rare combination of knowledge elements, even if each element has been known, the document can be considered to be novel. In this category, Boudreau *et al*. [9] measured the novelty of a research grant proposal based on a new combination of MeSH keywords. Similarly, drawing on a controlled dictionary of patent classifications, Verhoeven *et al*. [27] measured recombinant novelty by a new combination of IPC codes assigned to the patent. Arts *et al*. [25] also measured the novelty of a patent based on a new combination of two words that appeared in the patent.

The first and second groups are intuitively straightforward but have some limitations. Among others, these measures largely disregard semantic information included in text data. For example, the first group may consider a new synonym of an existing concept to be novel, unless controlled dictionaries are available. Similarly, the second group may consider any recombination equally novel regardless of the semantic distance between combined elements.

### (3) Recombination of cited documents

The third group also measures recombinant novelty, but instead of using text information, it draws on citation information. A document citing another document implies that knowledge in the latter is used by the former [28]. Thus, a document can be characterized by its cited documents, by considering each of cited documents to be a knowledge element that is incorporated into the citing document. Based on the recombinant novelty concept [18, 19], a document citing a set of documents that have rarely been cited together can be considered as a sign of novelty. In contrast to the first and second groups, in which a single word is considered a representation of knowledge, considering a cited document as a knowledge element adds semantic richness, making this approach popular in previous studies.

In this group, Dahlin and Behrens [16] proposed a novelty measure of patents based on a rare combination of cited references. Trapido [10] applied the same approach to journal articles, specifically in the field of electrical engineering. This approach is extended by Matsumoto *et al*. [17] so that it is applicable in any scientific field. A variation of this approach is to draw on journals in which cited documents are published [8, 11]. That is, if a focal document cites documents in two journals that have rarely been cited together, it is considered as a sign of novelty. This approach thus consolidates the unit of knowledge further at the journal level. Though considering a document or a journal as a unit of knowledge, without needing to scrutinize the content of documents, is convenient, its validity is under dispute [12, 13].

### (4) A distant text

The last group quantifies the uniqueness of a document based on text analysis, and relies on more recent development of natural language processing (NLP) to extract semantic information. In particular, drawing on the *word embedding* technique, Hain *et al*. [15] proposed a measure of patent novelty. Word embeddings map each word to a high-dimensional vector (i.e., a list of numbers). It allows us to quantify a semantic relationship between a pair of words by calculating the distance between the vectors–i.e., similar words have close vectors while dissimilar words have remote vectors. Hain *et al*. [15] assigned a vector to each patent by aggregating the vectors for a set of words that appear in the patent. Then, they calculated a distance between every pair of patents, with which a patent remote from any other patent is considered to be novel.

## Proposed measure of novelty

### Measuring novelty with word embedding

As a new approach, we propose to measure recombinant novelty of scientific documents by applying the combination of the word embedding technique and citation analysis. We consider a cited document as an appropriate unit of knowledge input, as in Group 3. Unlike the previous measures, which disregard the content of cited documents, we draw on the word embedding technique to extract semantic information in cited documents.

The word embedding technique often draws on machine learning algorithms (e.g., word2vec) to calculate a vector representation for each word based on the co-occurrences of words in a text corpus [22]. The approach is gaining confidence as the performance of machine learning has been improving, and has been recently applied to scientific documents for various purposes. For example, Tshitoyan *et al*. [29] captures the knowledge structure in the extant literature in material sciences with which they predict future scientific discoveries in the field. Still, to the best of our knowledge, the technique has not been used to measure the novelty of scientific documents.

Although computing word embeddings is demanding, some algorithms are publicly available, and some well-trained word embedding models (a list of vectors for a set of vocabularies) are also publicly accessible [30]. In this study, we use *scispaCy* as an established and publicly available library of word embeddings. ScispaCy builds on a popular *spaCy* model [30] and offers vector representations in a 200-dimensional vector space for 600,000 vocabularies specializing in biomedical texts [23, 31].

### Operationalization

With the selected word embedding library and citation information, the novelty of a document is computed through the following steps (Fig 1). Suppose that a focal document cites *N* references, and that each of the cited references has some text information. One can use various sources of text information, such as the full text and the abstract. In the following analysis, we construct respective measures from three text sources: the abstract, keywords, and the title of cited documents. Of the three sources, we intend to propose primarily using the title to minimize data requirement and maximize the utility of the measure.

**Step 1.** First, we vectorize the text information of the *i*-th reference as *v*_*i*_∈ℝ^200^ (*i*∈{1,…,*N*}). Since the text information includes multiple words, *v*_*i*_ is calculated as the mean of word embeddings of all words included.

**Step 2.** Second, we compute the distance of each pair of cited documents. The cosine distance between *i*-th and *j*-th references (1≤*i*<*j*≤*N*) is given by:

$$d_{ij}=1-\frac{v_{i}∙v_{j}}{\left|v_{i}||v_{j}\right|}$$ (1)

The cosine distance ranges from 0 to 2, where a larger value indicates a larger distance.

**Step 3.** Finally, we aggregate the distance scores over all pairs of cited references. In our dataset, one document has 32 cited references on average, which gives approximately 500 reference pairs. As a novelty measure of a focal document, we take the *q*-percentile value of the distance scores (*Novel*_*q*_), where *q*∈[0,100] and the 100-percentile value is defined as the maximum. Hence,

$$Novel_{q}=R^{-1}(\frac{Nq}{100})$$ (2)

where *R*(*d*_*ij*_) is the ordinal rank of *d*_*ij*_ of all the distances of *N*(*N*−1)/2 reference pairs.

### Computational cost

The aforementioned previous measures of novelty require extensive data access and processing. Text-based approaches (Table 1, Groups 1, 2, and 4) require the entire history of word uses, and citation-based approaches (Table 1, Group 3) need comprehensive citation network data. This poses two practical challenges for potential users of the novelty measures. First, the required data are usually proprietary, and thus, literally expensive. Second, processing the massive data takes high computational power. Not all users have such rich resources, compromising the utility of the measures.

Our proposed approach addresses these issues and aims to allow anyone to compute and use the novelty measures. Our measure requires only limited data access and little need for proprietary data. The measure can be computed only with the titles of a focal document’s cited references, which is often included in the focal document itself, and a publicly available library of word embeddings. The approach requires only small data processing. Unlike previous measures, our approach does not require extensive citation network analysis unlike Group 3, nor comparison with the whole document universe unlike Group 4. With the publicly shared code, anyone can compute the measure.

## Methods and data

Previous novelty measures have been rarely validated with a few exceptions [17]. To confirm the validity of our proposed measure, we carry out three exercises. The primary analysis is to test the criterion-related validity based on self-reported novelty scores for selected documents. As a preparatory step to this main analysis, we test whether scispaCy word embeddings can be indeed used to measure distances between documents (corresponding to Step 2). Finally, since novelty is known as a predictor of future citation impact [8, 11], we run regression analyses to test whether our proposed measure is positively associated with future citation.

To compute the proposed measures, we downloaded bibliometric information from Web of science (WoS). Since scispaCy specializes in the vocabularies in biomedicine, we focus on documents within relevant Subject Categories [32]. We focus on "article" as a document type and documents written in "English" [33]. We employ different sets of random samples for each analysis as detailed below.

### Validation of distance

Before validating the novelty measure itself, we test if scispaCy word embeddings convey semantic information of a text, and that they can assess the distance between a pair of documents. To this end, we compute distances of pairs of documents in two approaches–one based on scispaCy word embeddings and the other with a previously established approach–and confirm that the two are sufficiently correlated.

As a previously established approach, we compute the co-citation distance between a pair of documents *i* and *j*:

$$d_{ij}^{C}=1-\frac{coref_{ij}}{\sqrt{ref_{i}∙ref_{j}}}$$ (3)

where *ref*_*i*_ is the number of references cited by *i* and *coref*_*ij*_ is the number of references cited by both *i* and *j*. Co-citation distance has been previously used to measure the distance of scientific documents without a need to look into the content of the documents [10, 17]. A basic assumption is that a pair of documents should include a similar content if they cite a similar set of documents. We do not consider that the co-citation distance is superior to the word-embedding distance, but the two distances are expected to be correlated if scispaCy word embeddings do convey semantic information.

Second, using scispaCy word embeddings, we assign vectors respectively to the same pair of documents *i* and *j* (see Step 1 in Fig 1) and compute their distance (Eq 1). As text data for vectorization, we draw respectively on three sources (the title, the abstract, and keywords) from the pair of documents, preparing three distance measures ($d_{ij}^{T},\;d_{ij}^{A}$, and $d_{ij}^{K}$). Note that the word-embedding distance between a pair of focal documents is computed in this analysis, and this is applied to pairs of references cited by focal documents when we compute novelty.

For this analysis, we employed the following sampling strategy. First, we randomly sampled 100 authors in the field of biomedicine. Then, we collected all documents authored by these authors [34]. Finally, we filtered out documents outside of the biomedical field as well as documents missing reference information, resulting in 1,600 documents (16 documents per author on average). We compute the distance measures between documents written by the same author (i.e., we do not compare documents written by different authors). This is because co-citation is rare between a randomly chosen pair of documents written by different authors, which spuriously inflates the correlation.

### Validation of novelty

After confirming that the scispaCy word embeddings carry semantic information of text, we test the criterion-related validity of the proposed novelty measure (Eq 2). To this end, we draw on self-reported novelty scores, which we obtained from a questionnaire survey we conducted in 2009–2010 [35, 36]. The survey was responded by 2,081 scientists from various scientific fields, of whom this study draws on a subset of 321 respondents in biomedical fields.

The survey included a wide range of questionnaire items, one section of which asked the respondents to assess a randomly selected journal article that they published in 2001–2006. This section includes eight items to characterize the finding reported in the article (Table 2). As novelty is a multifaceted concept [37], the survey incorporated four aspects (theory, phenomenon, method, and material) in which the article may make scientific contribution. For each aspect, the survey further included two items, one indicating newness and the other indicating improvement over existing literature. We expect that the proposed measure should be correlated more with the newness items but less with the improvement items. Each item was responded in a 5-point scale (1: not relevant at all—5: highly relevant).

**Table 2:** Questionnaire of novelty.

Aspect	New vs. Improvement	Questionnaire item
Theory / Hypothesis	New	(1) Developing a new hypothesis or theory
	Improvement	(2) Supporting or rejecting an existing hypothesis or theory
Phenomenon	New	(3) Discovering an unknown phenomenon or material
	Improvement	(4) Understanding a phenomenon
Method	New	(5) Developing a new research method
	Improvement	(6) Improving an existing research method
Material / Function / Mechanism	New	(7) Creating a new function, mechanism, or material
	Improvement	(8) Improving on an existing function, mechanism, or material

Note. Responded in a 5-point scale (1: not relevant at all—5: highly relevant).

For the selected articles, we computed the proposed novelty measures (Eq 2), based on the title, the abstract, and keywords respectively, which generates three series of novelty measures ($Novel_{q}^{T},\;Novel_{q}^{A}$, and $Novel_{q}^{K}$) where *q*∈{100, 99, 95, 90, 80, 50}.

### Prediction of future citation

Previous studies consistently indicate a positive association between novelty and future citation impact of scientific documents [8, 11]. Thus, we test whether the proposed novelty measure can predict future citation effectively. For this analysis, we use "top-1% cited" (*TC*) in the respective field as the dependent variable and regress it on the proposed novelty measures. *TC* is a dummy variable coded 1 if the citation count of the article is within top 1% and 0 otherwise. Three sets of novelty measures are calculated with the title, the abstract, and keywords respectively ($Novel_{q}^{T},\;Novel_{q}^{A}$, and $Novel_{q}^{K}$) where *q*∈{100, 99, 95, 90, 80, 50}. Since the dependent variable is a dummy variable, we draw on logistic regressions:

$$\mathrm{Pr}\left(TC=1\right)=f(\beta_{0}+\beta_{1}Novel_{q}+\varepsilon )$$ (4)

where *f* is the logistic function.

For this analysis, we randomly sampled 2,000 articles published in biomedicine fields in 2010, and evaluated their citation impact as of 2020 (10 years after publication). We oversampled top-1% cited articles, so that the final sample consists of approximately 1,000 top-1% cited articles and 1,000 non-top-1% cited articles.

## Results

### Description of the measure

**Fig 2:**
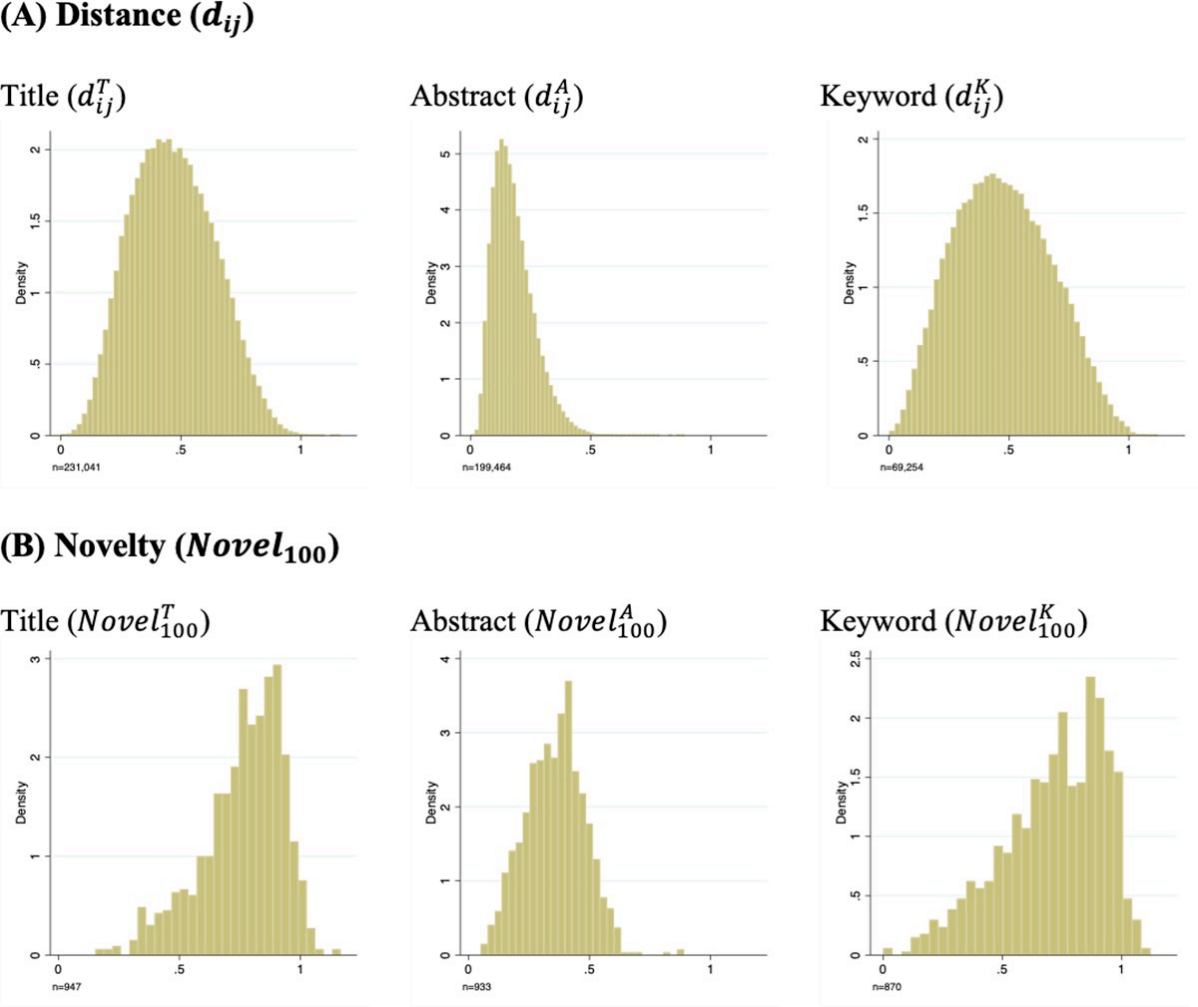
Distribution of distance and novelty. The same sample for the third validation study (prediction of future citation) is used, except that oversampled highly-cited documents are excluded. The 947 selected documents include in total approximately 230,000 combinations of cited references, for which the distance (Eq 1) is computed (A). The distances are summarized at the focal document level (Eq 2), and *Novel*_100_ is displayed as an example (B). Novelty measures with different *q* values are illustrated in S1 Appendix. Since abstracts and keywords are not available for all documents, the sample sizes are smaller.

To illustrate the distribution of the proposed measures, we computed the novelty of randomly selected documents (Fig 2B) and the distances of cited references of the documents (Fig 2A). Comparing distances based on three text data sources, Fig 2A shows that the abstract-based measure ($d_{ij}^{A}$) takes lower values. This is because abstracts include longer text information, which increases the chance that two cited documents share something in common. Based on the distances, novelty measures (*Novel*_*q*_) with various *q*’s are computed (see S1 Appendix). Fig 2B presents *Novel*_100_, which takes the maximum value of all reference pairs.

### Validation of distance

Table 3 presents the result of the validation of the distance measures. The sampled 1,600 articles authored by 100 authors yield 21,908 article pairs to compute the distances for. As the abstract and author keywords are not always available, the sample sizes are smaller for the analyses of the distances based on abstracts ($d_{ij}^{A}$) and keywords ($d_{ij}^{K}$).

**Table 3:** Validation of distance measures.

**(A) All distance measures**			
	Co-citation ($d_{ij}^{C}$)	Title ($d_{ij}^{T}$)	Abstract ($d_{ij}^{A}$)
Title ($d_{ij}^{T}$)	.231*** (21,908)		
Abstract ($d_{ij}^{A}$)	.310*** (16,706)	.337*** (16,706)	
Keyword ($d_{ij}^{K}$)	.318*** (8,781)	.450*** (8,781)	.407*** (8,481)
**(B) Co-citation distance ($d_{ij}^{C}$) and title distance ($d_{ij}^{T}$) by title length**			
Title word count	Correlation coefficient		
1–10	.440*** (1,395)		
11–13	.246*** (1,151)		
14–17	.197*** (1,827)		
18-	.259*** (1,867)		

Note. Pearson’s correlation coefficient (the number of observations in parentheses).

***p<0.001. (B) Subsamples of document pairs are selected based on the title word count of both paired documents.

Table 3A shows that three word-embedding distances ($d_{ij}^{T},\;d_{ij}^{A}$, and $d_{ij}^{K}$) are all strongly positively correlated with the co-citation distance ($d_{ij}^{C}$). Given that the co-citation distance is an accepted measure for the distance of scientific documents, this result supports our idea that scispaCy word embeddings can be used as a basis of novelty measurement. Compared to the title distance ($d_{ij}^{T}$), the abstract distance ($d_{ij}^{A}$) and the keyword distance ($d_{ij}^{A}$) indicate greater correlations with the co-citation distance ($d_{ij}^{C}$). Still, the title distance ($d_{ij}^{T}$) has a strongly significant correlation.

As we propose to use titles as the main source of text data for practical utility, we further examine the validity of the title distance ($d_{ij}^{T}$). We anticipated that short titles may carry insufficient semantic information and may not allow us to compute the distance reliably. We thus carry out correlation analyses with documents with different title lengths (word counts). Table 3B shows that the title distance ($d_{ij}^{T}$) is significantly correlated with the co-citation distance ($d_{ij}^{C}$) regardless of the word count. In fact, the correlation is strongest when the title length is shortest (10 words or shorter), contrary to our expectation. Thus, longer titles might bring more noise than information.

### Validation of novelty

**Fig 3:**
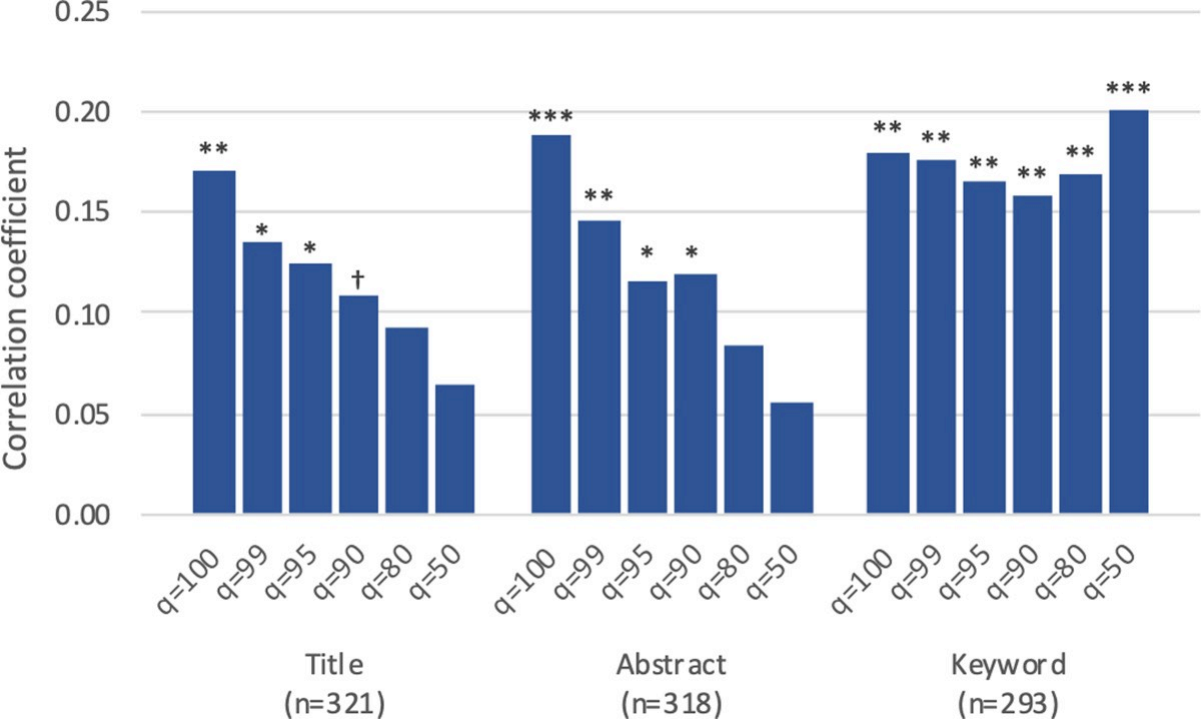
Correlation between bibliometric and self-reported novelty measures. Pearson’s correlation coefficient. *Novel*_*q*_ (*q*∈{100,99,95,90,80,50}) is correlated with the mean of four self-reported newness scores (Column 9 in Table 4). ^†^p<0.1, *p<0.05, **p<0.01, ***p<0.001.

Table 4 reports the correlation between the series of the proposed bibliometric measures (on the vertical axis) and the self-reported questionnaire scores (on the horizontal axis). On top of the eight scores from the questionnaire, we added two summary scores by taking the mean of the four newness scores (Column 9) and the mean of the four improvement scores (Column 10) respectively. We expect that our proposed measure should be correlated with the newness scores (Columns 1, 3, 5, 7, and 9) rather than the improvement scores (Columns 2, 4, 6, 8, and 10). Focusing on the newness summary score (Column 9), Fig 3 illustrates the correlation coefficients with novelty measures from three different text sources and with different *q* values.

**Table 4:** Validation of novelty measures.

			Self-reported measure																			
			Theory				Phenomenon				Method				Material				Summary			
			(1) New		(2) Impr.		(3) New		(4) Impr.		(5) New		(6) Impr.		(7) New		(8) Impr.		(9) New		(10) Impr.	
Bibliometric measures	Title ($Novel_{q}^{T}$)	*q* = 100	.126	*	-.012		.137	*	.066		.038		-.002		.187	***	.014	.170	**	.024	
		*q* = 99	.096	^†^	-.021		.097	^†^	.034		.017		-.015		.173	**	.016	.135	*	.004	
		*q* = 95	.086		-.038		.076		.031		.021		-.009		.174	**	.021	.125	*	.002	
		*q* = 90	.073		-.065		.060		.030		.028		-.010		.147	**	.004	.108	^†^	-.016	
		*q* = 80	.066		-.079		.048		.027		.022		-.017		.127	*	-.003	.092		-.027	
		*q* = 50	.051		-.082		.027		.024		.008		-.072		.098	^†^	-.019	.065		-.057	
	Abstract ($Novel_{q}^{A}$)	*q* = 100	.159	**	-.049		.130	*	.067		.038		-.034		.212	***	-.038	.188	***	-.022	
		*q* = 99	.131	*	-.074		.085		.040		.017		-.041		.184	**	-.043	.146	**	-.045	
		*q* = 95	.109	^†^	-.094	^†^	.058		.021		-.007		-.051		.169	**	-.050	.116	*	-.066	
		*q* = 90	.108	^†^	-.116	*	.059		.042		-.002		-.053		.173	**	-.066	.119	*	-.074	
		*q* = 80	.080		-.145	**	.053		.039		-.043		-.077		.142	*	-.089	.083		-.104	^†^
		*q* = 50	.019		-.162	**	.079		.041		-.074		-.104	^+^	.119	^†^	-.088	.055		-.120	*
	Keyword ($Novel_{q}^{K}$)	*q* = 100	.186	**	.068		.144	*	.136	*	.065		-.030		.129	*	.022	.180	**	.070	
		*q* = 99	.188	**	.059		.137	*	.139	*	.057		-.032		.125	*	.024	.175	**	.068	
		*q* = 95	.183	**	.043		.124	*	.128	*	.056		-.041		.118	*	.027	.165	**	.055	
		*q* = 90	.198	***	.022		.112	^†^	.117	*	.051		-.044		.103	^†^	.011	.158	**	.036	
		*q* = 80	.194	***	.009		.123	*	.087		.072		-.043		.104	^†^	.006	.168	**	.020	
		*q* = 50	.180	**	-.019		.164	**	.087		.118	*	-.025		.120	*	.020	.200	***	.022	

Note. Pearson’s correlation coefficient.

^†^p<0.1

*p<0.05

**p<0.01

***p<0.001. N = 321 (Title), 318 (Abstract), and 293 (Keyword).

The result presents a few findings mostly consistent with our expectation. First, Column 9 shows significant correlations between the proposed measures and the self-reported newness score, while Column 10 shows insignificant or negatively significant correlations with the self-reported improvement score. This suggests that our proposed approach does measure the newness of a scientific document and can distinguish novel discoveries from mere improvements. Second, comparing different *q* values, the result shows more positive correlation coefficients for the title and abstract measures ($Novel_{q}^{T}$ and $Novel_{q}^{A}$) with greater *q*’s. This suggests that a small number of distant recombination (even a single new combination), rather than many recombinations, is sufficient for a document to be novel. Interestingly, however, correlation coefficients for the keyword-based measures ($Novel_{q}^{K}$) are rather constant over a range of *q* values. Third, comparing the three sources of text information, the result overall shows somewhat larger correlation coefficients for the keyword-based measures ($Novel_{q}^{K}$) than for the abstract-based ($Novel_{q}^{A}$) and the title-based measures ($Novel_{q}^{T}$). Nonetheless, the difference is not substantial when we focus on the measures with the highest *q* (*Novel*_100_). The title-based novelty ($Novel_{100}^{T}$), which has the smallest correlation, is still strongly correlated with the self-reported newness summary score (*r* = .170, p < .01). Finally, looking into different aspects of newness and improvement (Columns 1–8), the result shows that newness in terms of theory, phenomenon, and material (Columns 1, 3, and 7) are correlated with the proposed measures but newness in terms of method (Column 5) is not. This may be attributed to a specificity of the biomedical field and needs further investigation.

### Prediction of future citation

Table 5 reports the result of logistic regressions to test if our proposed novelty measures predict future citation impact. It presents the odds ratios that a document falls within the top one percentile of citation counts. For example, the odds of a document with $Novel_{100}^{T}=1$ to be in the top one percentile is 154 times the odds of a document with $Novel_{100}^{T}=0$. Overall, the result shows significantly positive correlations between most variations of the novelty measures and citation impact, supporting the construct validity of our novelty measures.

**Table 5:** Odds ratio of top-1% citation rank.

	${Novel}_{q}^{T}$		${Novel}_{q}^{A}$		${Novel}_{q}^{K}$	
*q =* 100	154.02	***	27.89	***	5.91	***
*q =* 99	53.26	***	7.98	***	4.42	***
*q =* 95	29.27	***	3.17	*	3.46	***
*q =* 90	20.30	***	2.26		2.89	***
*q =* 80	14.97	***	1.04		2.04	**
*q =* 50	5.87	***	0.08	**	1.31	
N	1,921		1,903		1,814	

Note. Logistic regressions. Two-tailed test.

*p<0.05

**p<0.01

***p<0.001. The sampling weight is incorporated in the regression analysis.

**Fig 4:**
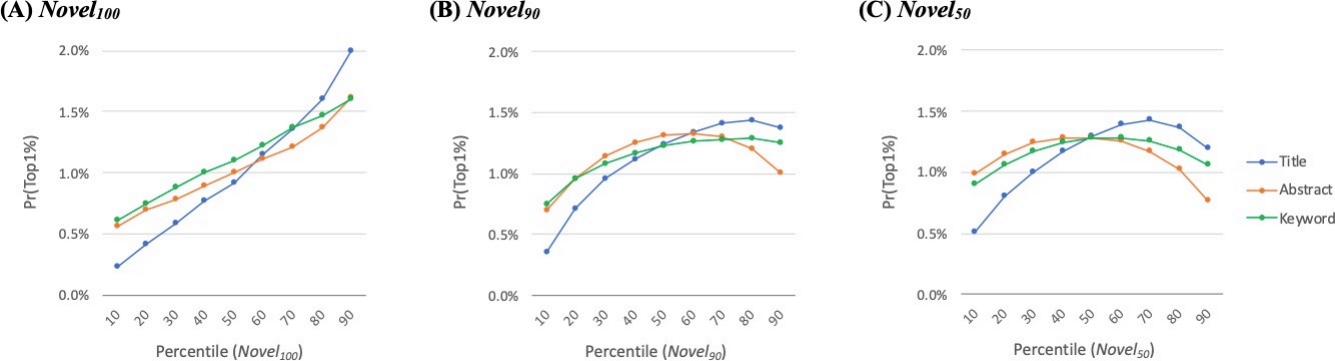
Prediction of top-1% citation rank. The probability of a focal document falling within the top 1 percentile is predicted. For easier interpretation and comparison, the horizontal axis takes the percentile of the novelty measures. (A) based on Row 1 in Table 5. (B) and (C) based on curvilinear models incorporating the quadratic term of the novelty measures (S1 Appendix).

The result also shows that the measures with greater *q*’s (e.g., *Novel*_100_) have higher odds ratios with greater statistical significance. This suggests that documents with a small number of distant recombination (even a single new combination) is sufficient to attract citations. Further to compare different text sources, Fig 4A graphically illustrates the regression result for *Novel*_100_. The graph shows that the title-based measure ($Novel_{100}^{T}$) has steeper curves than those based on abstracts ($Novel_{100}^{A}$) and keywords ($Novel_{100}^{K}$), thus best distinguishing highly-cited documents from less cited documents.

Since previous studies occasionally reported that novelty and citation impact have an inverted-U shaped relationship [38], we regress the citation impact on the quadratic term of the novelty measures on top of the linear term:

$$\mathrm{Pr}\left(TC=1\right)=f(\beta_{0}+\beta_{1}Novel_{q}+\beta_{2}{\left(Novel_{q}\right)}^{2}+\varepsilon )$$ (5)

We find that adding the quadratic term increases a model fit for the novelty measures with smaller *q*’s. Fig 4B and 4C illustrate the curvilinear associations for *Novel*_90_ and *Novel*_50_, showing that the optimal level of novelty scores decreases for lower *q*’s. This also suggests that a document with too many recombinations does not attract citation.

### Alternative measure of recombination within a document

Although the proposed measure utilizes recombination between cited documents, it is plausible to find recombination within a focal document itself. By decomposing the text information (the title, the abstract, or keywords) of a focal document into words, assigning word embeddings to them, and measuring the distance of every pair of words, we additionally constructed similar sets of novelty measures. This is in line with a category of previous measures [25] except that we use word embeddings to compute word distances.

We tested the validity of this additional set of measures for the correlation with self-reported novelty as well as for the prediction of future citation (S1 Appendix). The result is overall unsatisfactory. Correlations with the self-reported scores are mostly insignificant and sometimes negatively significant. Similarly, correlations with future citation impact are insignificant or negatively significant. Thus, the proposed approach to quantify recombinant novelty does not work with the text information within a focal document itself. This contrasts with the previous measures of recombination within a document [9, 25], which may be attributable to a different operationalization that the previous measures are based on the first appearance of a combined use of two words rather than their distance.

## Discussion and conclusion

Novelty is a core value in science [1, 2], and thus, a reliable approach to measure the novelty of scientific documents in a large scale is crucial. This study is the first to propose measuring the recombinant novelty of scientific documents based on the word-embedding technique. Most previous measures for recombinant novelty in science have been based solely on citation data [8, 10, 11, 16, 17]. Although citation network data is an effective tool to indirectly retrieve semantic information, recent advancement in text analysis allows us to extract it more directly and possibly more accurately [39, 40]. Combining citation data and text data, we provide a well-validated and user-friendly measure of scientific novelty.

One limitation common to most previous measures is insufficient validation [17]. To address this issue, we investigated our proposed measure from multiple angles. First, we show that the word embeddings, with which the novelty measure is computed, can be used to gauge the distance between scientific documents. Second, the novelty measures are significantly positively correlated with self-reported scores for various dimensions of newness but not with those for improvement, suggesting that the proposed measure can distinguish novel discoveries from mere improvements. Third, the novelty measure is found to be a significant predictor of citation impact in 10 years. Overall, these results confirm the validity of the proposed measure.

We examined several variations of novelty measures. First, we tested different percentile values (*q*) in aggregating the distance scores across all pairs of cited references. The result shows greater performance with higher *q*’s both in the correlation with self-reported novelty measures and in the prediction of future citation. Thus, the novelty of scientific documents is determined by a small number of distant recombination. This contrasts with the previous recombinant novelty measures based on more average distances [9].

Second, we use three different sources of text data, the abstract, keywords, and the title of cited references, to which the word-embedding technique is applied. The three text sources have different advantages. Abstracts offer rich information and keywords may be beneficial for conciseness, while titles are easiest to access. Based on the validation exercises, we find that the abstract-based measure ($Novel_{100}^{A}$), if we focus on the highest *q* = 100, demonstrates slightly higher performance in the correlation with the self-reported novelty scores, though the difference is only marginal. In the prediction of future citation, the title-based measure ($Novel_{100}^{T}$) presents highest performance. Overall, we recommend the title-based measure for data accessibility and reasonable validation results.

Another limitation common to previous measures is their computational cost for expensive data access as well as processing of massive data. Many potential users of the novelty measure cannot afford to it, which has substantially compromised the utility of the measures and delayed the progress of studies on scientific novelty. Our proposed approach overcomes these challenges. Drawing on limited text information (titles of cited references) and publicly shared library of word embeddings (scispaCy), our approach minimizes data access requirement as well as computational cost. Using the shared code, one can compute the novelty score of a document of interest only with the reference list of the document. Thus, we encourage the application of the approach for various purposes.

The approach has two limitations that future work needs to address. First, it depends on publicly available word-embedding libraries. ScispaCy specializes in biomedicine. Similar libraries are available in some fields but not in others, in which one needs to start with computing word embeddings. When a different library is used, the external validity of our approach needs to be tested. Second, we disregard the time dependency of word embeddings. The semantic distances between words change over time. Iterated computation of word embeddings may be required, for example, when novelty scores across different time points are to be compared.

## Supporting information

S1 Appendix Supplementary analysis. (PDF)

S1 Dataset (CSV)

S2 Dataset (CSV)

S3 Dataset (CSV)

S4 Dataset (CSV)
