# Correction: Measuring novelty in science with word embedding

**DOI:** 10.1371/journal.pone.0341474

**Published:** 2026-01-30

**Authors:** The PLOS One Staff

During the production process some cells were removed from Table 4. Please see the correct Table 4 here.

**Table 4:** Validation of novelty measures.

			Self-reported measure																		
			Theory				Phenomenon				Method				Material			Summary			
			(1) New		(2) Impr.		(3) New		(4) Impr.		(5) New		(6) Impr.		(7) New		(8) Impr.	(9) New		(10) Impr.	
Bibliometric measures	Title ($Novel_{q}^{T}$)	*q*= 100	.126	^*^	−.012		.137	^*^	.066		.038		−.002		.187	^***^	.014	.170	^**^	.024	
		*q*= 99	.096	^†^	−.021		.097	^†^	.034		.017		−.015		.173	^**^	.016	.135	^*^	.004	
		*q*= 95	.086		−.038		.076		.031		.021		−.009		.174	^**^	.021	.125	^*^	.002	
		*q*= 90	.073		−.065		.060		.030		.028		−.010		.147	^**^	.004	.108	^†^	−.016	
		*q*= 80	.066		−.079		.048		.027		.022		−.017		.127	^*^	−.003	.092		−.027	
		*q*= 50	.051		−.082		.027		.024		.008		−.072		.098	^†^	−.019	.065		−.057	
	Abstract ($Novel_{q}^{A}$)	*q*= 100	.159	^**^	−.049		.130	^*^	.067		.038		−.034		.212	^***^	−.038	.188	^***^	−.022	
		*q*= 99	.131	^*^	−.074		.085		.040		.017		−.041		.184	^**^	−.043	.146	^**^	−.045	
		*q*= 95	.109	^†^	−.094	^†^	.058		.021		−.007		−.051		.169	^**^	−.050	.116	^*^	−.066	
		*q*= 90	.108	^†^	−.116	^*^	.059		.042		−.002		−.053		.173	^**^	−.066	.119	^*^	−.074	
		*q*= 80	.080		−.145	^**^	.053		.039		−.043		−.077		.142	^*^	−.089	.083		−.104	^†^
		*q*= 50	.019		−.162	^**^	.079		.041		−.074		−.104	^+^	.119	^†^	−.088	.055		−.120	^*^
	Keyword ($Novel_{q}^{K}$)	*q*= 100	.186	^**^	.068		.144	^*^	.136	^*^	.065		−.030		.129	^*^	.022	.180	^**^	.070	
		*q*= 99	.188	^**^	.059		.137	^*^	.139	^*^	.057		−.032		.125	^*^	.024	.175	^**^	.068	
		*q*= 95	.183	^**^	.043		.124	^*^	.128	^*^	.056		−.041		.118	^*^	.027	.165	^**^	.055	
		*q*= 90	.198	^***^	.022		.112	^†^	.117	^*^	.051		−.044		.103	^†^	.011	.158	^**^	.036	
		*q*= 80	.194	^***^	.009		.123	^*^	.087		.072		−.043		.104	^†^	.006	.168	^**^	.020	
		*q*= 50	.180	^**^	−.019		.164	^**^	.087		.118^*^		−.025		.120	^*^	.020	.200	^***^	.022	

The publisher apologizes for the error.
