# Supplementary material for: Measuring novelty in science with word embedding
Source: PLoS One. 2021 Jul 2;16(7):e0254034. doi: 10.1371/journal.pone.0254034 (PMC8253414; doi:10.1371/journal.pone.0254034)
Supplement: S1 Appendix — (PDF) [file pone.0254034.s001.pdf]

S1 Appendix. Supplementary Analysis

S1.1 Distribution of Measures

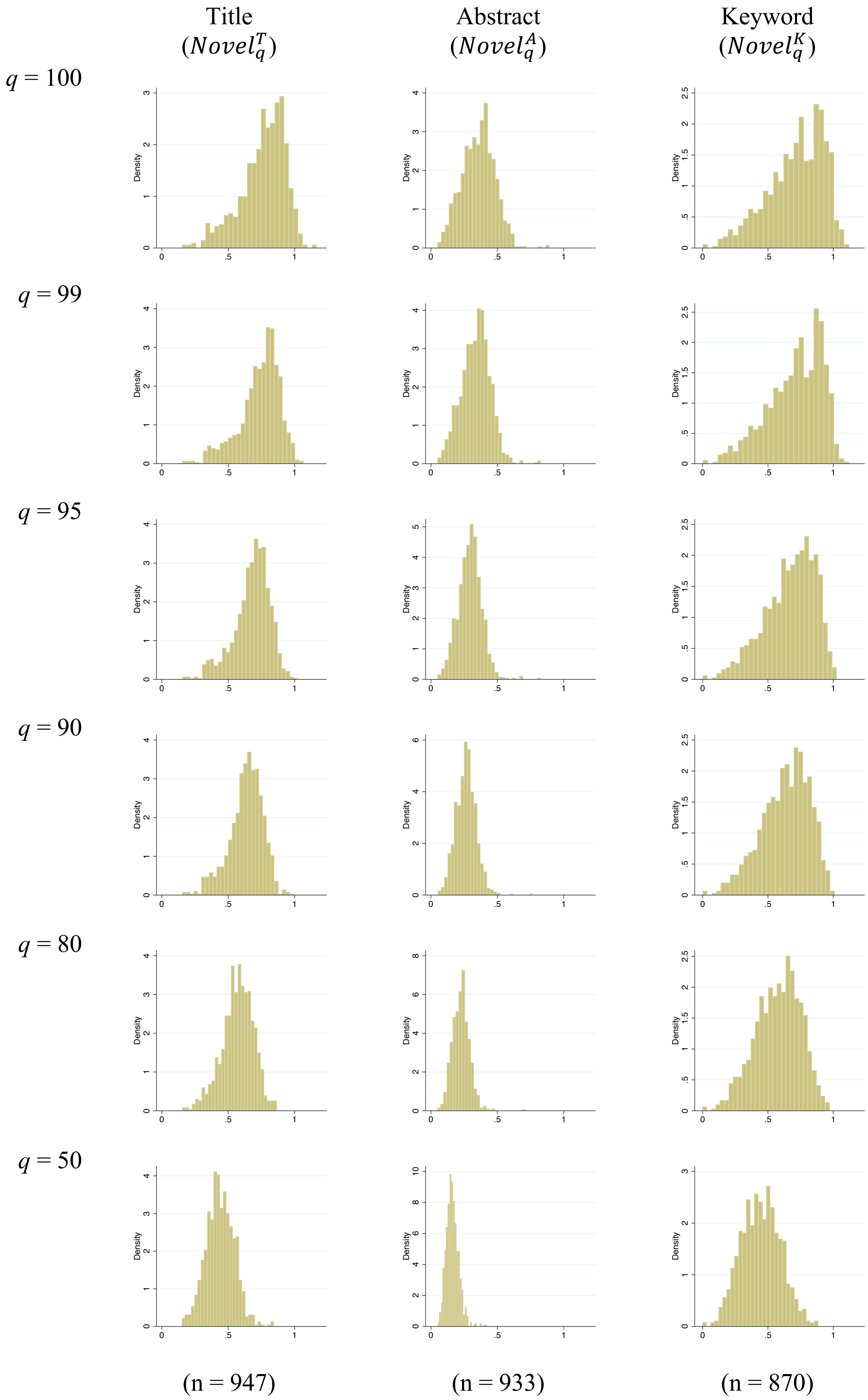

S1.2 Prediction of Top-1% Citation Rank (Curvilinear Model)

		$Novel_q^T$	$Novel_q^A$	$Novel_q^K$
q = 100	Linear ($\hat{\beta}_1$)	3.50	10.45***	-0.46
	Quadratic ($\hat{\beta}_2$)	0.95	-8.49***	1.61†
q = 99	Linear ($\hat{\beta}_1$)	17.10***	20.26***	2.04
	Quadratic ($\hat{\beta}_2$)	-8.60**	-25.07***	-0.41
q = 95	Linear ($\hat{\beta}_1$)	21.88***	23.25***	4.10**
	Quadratic ($\hat{\beta}_2$)	-13.25***	-35.88***	-2.23†
q = 90	Linear ($\hat{\beta}_1$)	26.68***	26.78***	5.11**
	Quadratic ($\hat{\beta}_2$)	-18.07***	-47.63***	-3.29*
q = 80	Linear ($\hat{\beta}_1$)	29.35***	25.15***	6.89***
	Quadratic ($\hat{\beta}_2$)	-22.54***	-54.05***	-5.44***
q = 50	Linear ($\hat{\beta}_1$)	29.01***	36.39***	6.82***
	Quadratic ($\hat{\beta}_2$)	-29.40***	-117.78***	-7.21***
Observation		1,921	1,903	1,814

Note. Logistic regressions. Two-tailed test. *p<0.05, **p<0.01, ***p<0.001. Unstandardized coefficients of the linear term ($\hat{\beta}_1$) and quadratic term ($\hat{\beta}_2$) are presented. The sampling weight is incorporated in the regression analysis.

S1.3 Validation of Novelty Measures (recombination within a document)

			Self-reported novelty score									
			Theory		Phenomenon		Method		Material		Total	
			New	Impr.	New	Impr.	New	Impr.	New	Impr.	New	Impr.
Bibliometric measures	Title ($Novel_q^T$)	q = 100	-.002	.013	.069	.083	-.008	.018	.108*	.107†	.062	.083
		q = 99	.000	-.005	.059	.043	-.030	.003	.099†	.109*	.048	.057
		q = 95	-.023	-.019	.023	.019	-.015	-.001	.064	.082	.020	.031
		q = 90	-.067	-.029	-.006	-.016	-.080	.005	.025	.058	-.040	.008
		q = 80	-.035	.031	-.009	-.013	-.087	.026	.033	.076	-.030	.046
		q = 50	-.073	.024	-.051	-.044	-.103†	-.059	-.058	.005	-.096†	-.029
	Abstract ($Novel_q^A$)	q = 100	.025	-.060	-.046	-.023	-.169**	-.097†	-.122*	-.069	-.108†	-.095†
		q = 99	-.026	-.092	.042	-.030	-.213***	-.174**	-.018	-.002	-.067	-.114*
		q = 95	-.075	-.034	.020	-.034	-.198***	-.114*	.005	.044	-.077	-.053
		q = 90	-.076	-.018	.013	-.057	-.205***	-.103†	-.004	.045	-.085	-.050
		q = 80	-.052	.024	-.003	-.033	-.192***	-.069	-.007	.058	-.081	-.008
		q = 50	-.060	-.018	-.025	-.016	-.188***	-.065	-.027	.083	-.097†	-.006
	Keyword ($Novel_q^K$)	q = 100	.024	-.026	.077	.043	.003	.088	.074	.090	.063	.078
		q = 99	.024	-.026	.077	.043	.003	.088	.074	.090	.063	.078
		q = 95	.013	-.036	.071	.034	.000	.082	.072	.083	.055	.066
		q = 90	.003	-.047	.050	.009	-.030	.086	.077	.088	.037	.056
		q = 80	-.066	.020	.046	-.024	-.131†	.007	.025	.044	-.037	.019
		q = 50	-.025	.041	.003	-.010	-.147*	-.002	.049	.078	-.036	.041

Note. Pearson's correlation coefficient. $^{\dagger}p<0.1$, $^{*}p<0.05$, $^{**}p<0.01$, $^{***}p<0.001$. N = 330 (Title), 311 (Abstract), and 205 (Keyword).

S1.4 Odds Ratio of Top-1% Citation Rank (recombination within a document)

	$Novel_q^T$		$Novel_q^A$		$Novel_q^K$	
q = 100	0.70		8.74	*	1.18	
q = 99	0.88		0.02	†	1.16	
q = 95	1.23		0.00	***	1.02	
q = 90	1.43		0.00	***	0.90	
q = 80	0.56		0.00	***	0.80	
q = 50	0.09	***	0.00	***	0.45	†
Observation	1,998		1,924		1,217	

Note. Logistic regressions. Two-tailed test. †p<0.1, *p<0.05, **p<0.01, ***p<0.001. The sampling weight is incorporated in the regression analysis.
